# The Role of Reactive Oxygen Species in Plant Response to Radiation

**DOI:** 10.3390/ijms24043346

**Published:** 2023-02-08

**Authors:** Yuantao Tan, Yaoke Duan, Qing Chi, Rong Wang, Yue Yin, Dongjie Cui, Shuang Li, Aiying Wang, Ruonan Ma, Bing Li, Zhen Jiao, Hao Sun

**Affiliations:** 1 Zhengzhou Research Base, State Key Laboratory of Cotton Biology, School of Agricultural Sciences, Zhengzhou University, Zhengzhou 450001, China; 2 Henan Key Laboratory of Ion-Beam Bioengineering, School of Agricultural Sciences, Zhengzhou University, Zhengzhou 450052, China; 3 Sanya Institute, Zhengzhou University, Zhengzhou 450001, China

**Keywords:** UV radiation, ion beam, plasma, reactive oxygen species, plant responses

## Abstract

Radiation is widespread in nature, including ultraviolet radiation from the sun, cosmic radiation and radiation emitted by natural radionuclides. Over the years, the increasing industrialization of human beings has brought about more radiation, such as enhanced UV-B radiation due to ground ozone decay, and the emission and contamination of nuclear waste due to the increasing nuclear power plants and radioactive material industry. With additional radiation reaching plants, both negative effects including damage to cell membranes, reduction of photosynthetic rate and premature aging and benefits such as growth promotion and stress resistance enhancement have been observed. ROS (Reactive oxygen species) are reactive oxidants in plant cells, including hydrogen peroxide (H_2_O_2_), superoxide anions (O_2_^•−^) and hydroxide anion radicals (·OH), which may stimulate the antioxidant system of plants and act as signaling molecules to regulate downstream reactions. A number of studies have observed the change of ROS in plant cells under radiation, and new technology such as RNA-seq has molecularly revealed the regulation of radiative biological effects by ROS. This review summarized recent progress on the role of ROS in plant response to radiations including UV, ion beam and plasma, and may help to reveal the mechanisms of plant responses to radiation.

## 1. Introduction

Radiation refers to the emission of electromagnetic energy away from the source, such as UV radiation, ion beams and plasma. It is classified as ionizing radiation or non-ionizing radiation according to its energy level and the ability to ionize materials [1,2]. Plants on earth are inevitably exposed to UV rays from sunlight and ionizing radiation from cosmic space or radionuclides in rocks and soils [3,4,5]. Moreover, human activities such as nuclear power generation, nuclear accidents, the storage of radioactive waste and operations associated with the naturally occurring radioactive material (NORM) industry lead to more radiation [5]. The effect of radiation on plants may be distinctive according to plant species or genotypes, radiation types and doses [6,7,8]. Generally, radiation has a negative effect on the growth, development, reproduction, metabolic activities and DNA integrity of plants. Nevertheless, some studies have indicated that weaker radiation may provide advantages to plant growth, metabolism, resistance to biotic stresses and quality [9,10,11].

Different wavelengths of the radiation spectrum can interact directly with macromolecules [1,12]; UV and ionizing radiation may also exert biological effects indirectly through reactive oxygen species (ROS) produced in plants [13,14,15,16]. ROS are a group of extremely reactive oxidants, including hydrogen peroxide (H_2_O_2_), superoxide anions (O_2_^•−^), and hydroxide anion radicals (·OH) [17]. These oxidants may function as signaling molecules to regulate plant growth and development, and control metabolite synthesis and stress responses [18]. The balance between ROS production and scavenging is critical because excessive ROS may bring oxidative stress on plants [18,19]. Previous research has observed that ROS production induced by UV radiation can obviously lead to lipid peroxidation and elevated malondialdehyde (MDA) accumulation in grains [6], as well as reduced photosynthetic efficiency in olive trees [20,21]; Additionally, ROS production has been linked to the mechanism of UV or other ionizing radiation-induced DNA damage [11,22,23]. In our previous research, the growth of *Arabidopsis* seedling roots after low-energy N^+^-beam treatment was obviously promoted, with an elevated accumulation of H_2_O_2_ and O_2_^•−^ and enhanced expression of respiratory burst oxidase homologs genes (*RBOHs*) [7], suggesting that ROS played a regulatory role in the promotion of *Arabidopsis* seedlings injected with low-energy N^+^-beam. In a different study, we also showed that the improved activity of the antioxidant system played an important role in the promotion of *Arabidopsis* seedling growth under low-dose carbon ion beam radiation [15]. All the above suggested that ROS may be involved in the biological effects of radiation on plants.

To relieve the deleterious effects of excess ROS, plant cells can remove them with an antioxidant system consisting of both enzymatic and non-enzymatic antioxidants [6,24,25]. The enzyme system mainly includes superoxide dismutase (SOD), catalase (CAT), peroxidase (POD), ascorbate peroxidase (APX), glutathione peroxidase (GPX) and glutathione S-transferase (GST) [24,25]. The non-enzymatic system is mainly mediated by low molecular weight antioxidants, such as glutathione (GSH), ascorbic acid (AsA) and flavonoids, which are known to remove hydroxyl radicals and singlet oxygen [26]. It was observed that after radiation-induced ROS imbalance, plant cells may relieve the ROS disruption through the systems or pathways above. Mishra et al. and Ozgur et al. found that ROS and the activity of antioxidant enzymes (SOD, CAT, APX, etc.) were increased in bitter gourd (*Momordica*. *charantia*), *Arabis alpina* and *Arabidopsis thaliana* after UV radiation [13,27], and similar results were observed in *Arabidopsis* and *Daucus carota* after ion beam or plasma treatment [15,28]. The elevated antioxidant system may participate in scavenging excess ROS.

With the help of bioinformatic approaches such as high throughput sequencing and molecular biological methods, new progress regarding the change and regulation of ROS in plant responses to radiation has been reported, especially regarding UV, ion beam and plasma treatments [7,22,29,30]. Table 1 presents a compilation of noteworthy reports that have explored the role of ROS in plants in response to different radiation sources. This review summarized recent progress concerning the role of ROS in plant responses to radiation, and may help to reveal the mechanisms of radiation’s biological effect on plants.

## 2. The Role of ROS in Plant Responses to UV Radiation

Most wild plants grown on earth acquire energy from sunlight. This energy is essential for photosynthesis, which occurs in the spectrum of 400–700 nm. Solar radiation also includes ultraviolet (UV) radiation, such as UV-A (315–400 nm), UV-B (280–315 nm) and UV-C (100–280 nm). The high energy and mutagenic UV-C is filtered out by the stratospheric ozone, so it does not reach the biosphere. Even though most UV-B is also blocked, the remaining radiation can still damage plant cells and affect physiological processes such as photosynthesis. The redox status, which is determined by ROS, may be involved in this process [54,55,56,57,58,59].

### 2.1. UV Radiation May Induce ROS Production and Activates Plant Antioxidant Systems

Exposure to excessive UV-B radiation can induce the production of ROS in plants [60,61,62,63], leading to an increase in malondialdehyde (MDA) accumulation. Plants have evolved a range of defense mechanisms against ROS produced in response to UV radiation stress. These mechanisms include antioxidant enzymes, such as CAT, POD, APX, glutathione reductase (GR) and SOD, as well as non-enzymatic systems such as ASA, GSH, flavonoids and alpha-tocopherol [6,25,59,64,65]. The catalytic activities of both enzymatic and non-enzymatic antioxidants in plants are demonstrated in Table 2. The degree of antioxidant response to UV radiation is dependent on the duration, intensity and genotype of the plant. MDA is a product of ROS-mediated polyunsaturated membrane lipids and is often used as a marker of ROS [66,67]. Hideg et al. observed an increase in MDA levels in broad bean leaves exposed to 0.46 kJ m^−2^ UV-B radiation for a short period of time, indicating a change in free radicals [68]. Dai et al. found that UV-B radiation caused an increase in MDA and H_2_O_2_ concentrations in rice (*Oryza sativa*), as well as an increase in the rate of O_2_^•−^ generation, accompanied by membrane leakage and cell damage [69]. Kalbin et al. observed a significant increase in ROS levels in pea (*Pisum sativum*) tissues after strong UV-B exposure by measuring oxidized and total glutathione (GSSG and GSH) [70]. The redox state of antioxidants can be used to infer changes in ROS concentrations, as demonstrated by Mishra et al. (2008), who employed the method of Elstner et al. to estimate O_2_^•−^ by monitoring the formation of nitrite in the supernatant of seedling leaf homogenates of UV-B by hydroxylamine in the presence of O_2_^•−^ [13,34]. Progress in molecular biology and physics have enabled researchers to directly detect ROS accumulation. Barta et al. observed ROS in spinach leaves after UV-B radiation using fluorescent probes [71]. Hideg and Vass used EPR spectra analysis to detect free radical formation in UV-B irradiated broad bean leaves [72]. Similarly, Kurdziel et al. also discovered changes in ROS levels in wheat, oats, and barley [6]. Technological advances have enabled us to investigate the correlation between ROS and UV-B radiation in plants with greater ease and speed.

ROS production in UV-B radiation-treated plants may be associated with both metabolization response and photophysical reaction. Metabolic disturbances, such as impaired electron transfer in photosystem II (PSII) and other quinone components, can lead to ROS production in photosystem I (PSI) [73,74].

2O_2_ + 2Fd_red_ → 2 O_2_^•−^ + 2Fd_ox_

Additionally, UV-B radiation can convert H_2_O_2_ to highly reactive hydroxyl radicals (·OH) through photoconversion, which can increase oxidative damage [75].

2 O_2_^•−^ + 2H^+^ → H_2_O_2_ + O_2_

H_2_O_2_ + O_2_^•−^ → OH^-^ + O_2_ + ·OH

Furthermore, UV-induced ROS production can be caused by increased mitochondrial respiration and water ionization [76,77].

Studies have shown that UV-B radiation can induce the activity of enzymes such as SOD in *Arabidopsis* and other plants [78,79], and that this change can be time-sensitive. For instance, Rengin et al. observed that SOD activity decreased in both *Arabis alpina* and *Arabidopsis thaliana* after 3 h of UV-B irradiation, but was significantly increased after 6 h, indicating the activation of an adaptive response [27]. Studies have demonstrated that different plants exhibit distinct antioxidant responses when exposed to UV radiation. For instance, Giarraffa plants exposed to UV-B showed no significant changes in CAT and SOD activity, an increase in GPox activity, and a decrease in total flavonoid levels. Meanwhile, *Olivastra Seggianese* plants experienced a statistically different CAT response, an increase in SOD activity, a decrease in GPox activity, and high flavonoid levels until the end of the UV treatment [21]. Additionally, Kurdziel et al. observed that UV treatment increased SOD, APX, and GR activity in seed embryos of wheat, oats, and barley, but decreased SOD activity in their endosperm [6].

It has been suggested that ROS may act as a signaling molecule to regulate changes in the antioxidant system in response to UV-B radiation [80,81]. Studies have demonstrated that the application of specific H_2_O_2_ and O_2_^•−^ scavengers, as well as a general free radical scavenger, can inhibit UV-B-induced enzymatic antioxidant activity and non-enzymatic antioxidant regeneration in UV-B-treated *Neoporphyra haitanensis* [81]. Furthermore, it has been observed that chloroplast APXs are inhibited in the presence of H_2_O_2_ when ascorbic acid levels are reduced [82]. These findings suggest that UV-B radiation may cause ROS-mediated alterations in antioxidant status. However, further research is needed to elucidate the molecular mechanism of ROS regulation of the antioxidant system under UV-B radiation.

### 2.2. UV May Affect Metabolites Production via ROS

Exposure to UV-B radiation has been shown to induce the production of ROS and a wide range of metabolites such as ascorbic acid, β-carotene, anthocyanins, phenols, flavonoids, terpenoids, and alkaloids [40,83,84,85,86,87,88]. It has been found that the combination of wounding and UV radiation induced synthesis of various signaling compounds in carrot tissue [31]. The skin/cuticle of the plant contains UV-filtering compounds; thus, the amount of UV-induced ROS production is tissue-dependent. When wounding happens prior to UV radiation the skin/cuticle is partially removed, which increases the penetration area of the radiation, leading to increased ROS production as the primary signal for UV and as a signal for ethylene (ET) biosynthesis, upregulating the S-adenosyl-methionine synthetase 1 (SAM synthetase) gene. ET, in turn, activates jasmonic acid (JA) biosynthesis by upregulating Lipoxygenase 5 (LOX 5) and 12-oxophytodienoate reductase (12-OPDA reductase) genes, resulting in increased ET and JA production, further increasing phenylalanine ammonia-lyase (PAL) activity and phenolic accumulation [31,76,89].

Hydrogen peroxide is a ubiquitous signaling molecule in plant cells [90], and exogenous H_2_O_2_ has been shown to increase the content of isoflavones and anthocyanins in germinated soybean and radish sprouts [29,32]. Given that UV-B radiation induces H_2_O_2_ production, it is likely that H_2_O_2_ plays a role in conveying UV-B-induced signaling to downstream defense responses and in the upregulation of key enzymes involved in isoflavone biosynthesis [29]. Phenylalanine ammonia-lyase (PAL), chalcone synthase (CHS) and isoflavone synthase (IFS) are three of the key enzymes involved in isoflavone biosynthesis, and Ma et al. observed that H_2_O_2_ production resulting from UV-B exposure upregulated the activity, gene, and protein expression of these enzymes [29,91]. Further research is needed to ascertain if more genes or proteins are involved in the H_2_O_2_-mediated signaling transduction pathway. Regarding that, the radiation-induced production of ROS in plants to regulate the production of metabolites is relatively rare, and some authors suggest that H_2_O_2_ produced by UV-irradiated plants activates protein expressions of isoflavone biosynthesis-related enzymes [29]. Like other plant secondary metabolism alkaloids, terpenes, carotenoids, and thioglucosides, the relationship with ROS is still shallowly studied and these are crucial for plant defense systems to be further explored by subsequent authors.

### 2.3. UV May Affect Photosynthesis via ROS

The effects of ultraviolet-B (UV-B) radiation on photosynthetic organisms are well studied. Exposure to UV-B has been shown to reduce plant photosynthetic activity, likely due to the accumulation of ROS. These ROS can lead to lipid peroxidation, causing damage to the thylakoid membrane structure and disrupting the metabolic environment needed for photosynthesis [3,12,92,93]. Additionally, UV-B radiation has been observed to increase ion permeability in thylakoid, plasma and cultured cell membranes [94,95,96,97].

Photosystem II (PS II) is a pigment-protein complex embedded in the thylakoid membrane, which captures sunlight and converts it into chemical energy [3]. According to Swarna et al., UV-B radiation may reduce PS II photochemical efficiency up to 68% in maize leaves, which is attributed to an increase in ROS [33]. It is believed that the UV light is absorbed by the manganese (Mn) cluster in the oxygen-evolving complex (OEC) of PS II and causes primary photodamage [98,99,100]. Lidon et al., Hideg et al. and Szilard et al. have observed that ·OH accumulates after UV-B treatment, which is considered one of the possible mechanisms of UV-B-induced damage [72,101,102]. The changes in photosynthetic pigments under UV-B stress have been less studied. However, in *Eucalyptus globulus* and olive trees exposed to UV-B BED at approximately 6 and 12 KJ m^−2^ d^−1^, respectively, the increase in ROS was associated with a decrease in pigmentation in UV-B treated plants [35,36]. Piccini et al. found that in the Giarraffa variety UV-B seems to reduce the accumulation of pigments (chlorophylls and β-carotene), particularly after a prolonged period of UV-B exposure [20].

Ultraviolet-B (UV-B) radiation has been observed to induce damage in plant leaves, particularly in proteins such as Rubisco. Research has suggested that UV-B-generated ROS may be responsible for the degradation of Rubisco via the proteolytic degradation of large subunits (LSU) [37,38,39]. This has been further supported by studies which have found that ROS can directly cleave LSU in chloroplasts under light conditions [103].

In conclusion, UV-B radiation can lead to oxidative damage to membrane lipids and proteins, resulting in decreased photosynthetic pigment and protein content, lower PS II photosynthetic efficiency, and lower Rubisco activity. This ultimately leads to reduced crop yield.

### 2.4. ROS May Be Involved in the Regulation of Gene Expression under UV Radiation

**Figure 1:**
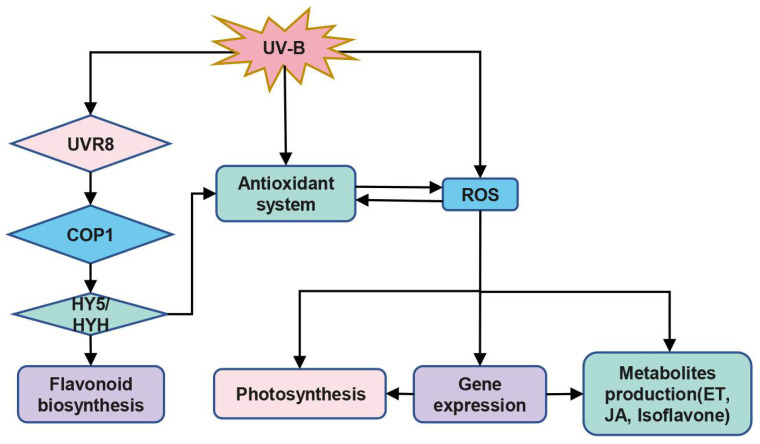
A simple mechanism for plant response to UV-B. ROS, reactive oxygen species; UVR8/COP1, regulators of UV-B responses; HY5/HYH, transcription factors; ET, ethylene; JA, jasmonic acid.

Under low (2~8 KJ m^−2^ d^−1^) and high (>8 KJ m^−2^ d^−1^) doses of UV-B radiation, plants employ different signaling pathways [104]. Low doses of UV-B radiation stimulate the rapid translocation of UVR8 protein from the cytoplasm to the nucleus, activating and interacting with constitutively photomorphogenic 1 (COP1), a key regulator of light signaling (Figure 1). This interaction triggers the expression of *elongated hypocotyl 5* (*HY5*) and *HY5* homolog (*HYH*) [86], as well as the activation of flavonoid biosynthesis and antioxidant defense [105,106,107]. The molecular mechanism of plant response to high doses of UV-B radiation is largely unknown, but several studies have suggested that ROS may be involved. For example, Mackerness et al. demonstrated that O_2_^•−^ was significantly increased in *Arabidopsis* after UV-B radiation, which may induce the expression of *PDF1.2* [40]. Additionally, H_2_O_2_ produced by O_2_^•−^ disproportionation may increase the expression of *PR-1* but inhibit *Lhcb* [40].

## 3. The Function of ROS in Plant Response to Ion Beam

It is widely accepted that both natural and anthropogenic sources of ionizing radiation expose the environment to radiation [23,108,109]. Ion beam irradiation, due to its high energy and strong penetrative capacity, is a significant form of environmental ionizing radiation, which can directly interact with matter to cause ionization and excitation. The absorption of energetic ions by biological materials can lead to changes in physical, physicochemical, and chemical processes, which can endow modern plants with advantageous agronomic features and microbiological resistance [110,111]. Additionally, plants may react to ion injection by generating free radicals, causing oxidative stress, altering signal transduction, and even causing death [41,112,113,114].

### 3.1. Ion Beam May Enhance Plant Stress Resistance by Modulating ROS Levels

**Figure 2:**
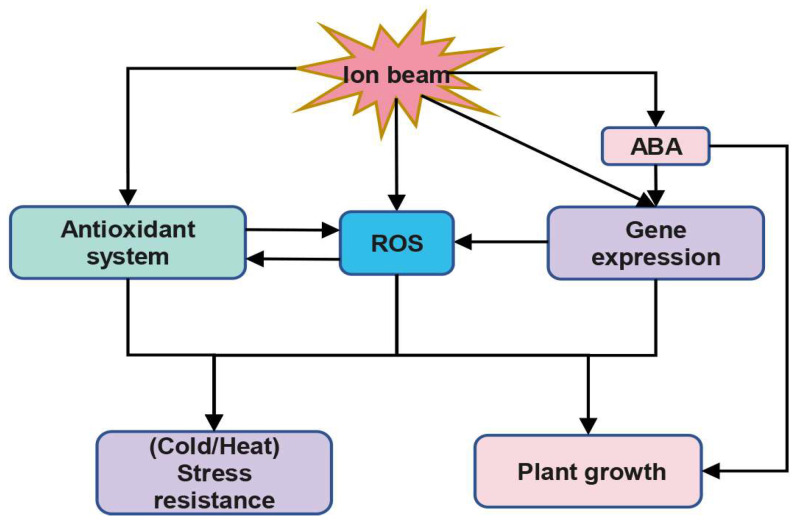
A simple mechanism for plant response to ion beam. ROS, reactive oxygen species; ABA, abscisic acid.

Ion beam radiation has been demonstrated to improve plant resistance and reduce growth stress under cold or heat stress (Figure 2) [41,42]. Wang et al. found that 50-Gy carbon ion beam irradiation increased cold tolerance in *Arabidopsis thaliana*, leading to a decrease in the levels of oxidative stress indicators (H_2_O_2_ content, O_2_^•−^ production rate, ·OH production activity and MDA content) and an increase in SOD and CAT activities [42]. Similarly, Wang et al. showed that carbon ion beam irradiation increased the heat resistance of *Arabidopsis* seedlings by activating several pathways, such as proline accumulation, antioxidant enzymes, and the AsA-GSH cycle, thereby reducing the effects of oxidative stress damage [41]. Several cold regulated genes including C-repeat-binding factor (*CBF*), C-repeat-binding factor expression 1 (*ICE1*), *ICE2*, calmodulin-binding transcription activator 3 (*CAMTA3*), R2R3 type transcription factor *MYB15* and C2H2 zinc-finger protein *ZAT12*, as well as cold responsive (*COR*) genes such as *COR15a* and *COR15b*, and heat related genes, such as *Hsp70*, *Hsp18.2* and *P5cs*, were found to be significantly altered in *Arabidopsis thaliana* seedlings exposed to 50-Gy carbon ion beam irradiation under low or high temperature stress [42,115,116,117,118]. These genes are associated with heat stress resistance, proline scavenging of free radicals, and proline synthesis and degradation, respectively [119,120,121]. It has also been demonstrated that H_2_O_2_, O_2_^•−^ and NO produced by ion beam will act as excitation signals for disease resistance factors in wheat and improve the disease resistance of wheat [43]. It remains to be seen whether ROS affect the expression of these genes.

### 3.2. Ion Beam May Affect Plant Growth via ROS

Research has shown that ion beam irradiation has diverse effects on the growth of plant seedlings, depending on the dose. Low doses of the carbon ion beam have been demonstrated to have a stimulating effect on growth parameters such as plant height, root length and fresh weight of rice [122,123]. Sjahril et al. found that treating rice seeds with argon ion irradiation was more advantageous to seedling growth than carbon ion irradiation [124]. Wang et al. found that a dose of 50 Gy of carbon ion beam irradiation had a growth-promoting effect on *Arabidopsis* seedlings, with a significant increase in ROS (O_2_^•−^ production rate, ·OH producing activity, H_2_O_2_ content) levels and MDA content [15]. In contrast, high doses of ion beam irradiation have been widely shown to inhibit seedling growth, with studies observing dose-dependent growth inhibition in rice seedlings irradiated with carbon ion beams [112]. Additionally, Vilaithong et al. and Apavatjrut et al. found that high doses of Ar- or N-ion bombardment retarded the germination and growth of naked corn embryos [125,126]. Similarly, Yang et al. observed significant growth inhibition in *Arabidopsis* primary root elongation, sprouting and survival under a dose of 1.5107 ions cm^−2^, while Ya et al. observed dose-dependent growth inhibition in rice seedlings under low-energy N^+^ beam injection [44,113]. Research conducted by Yuqi Li et al. showed that the use of ^12^C and ^7^Li ion beams on winter wheat (*Triticum aestivum*) seedlings led to a decrease in their seedling height and root length [127]. This was further corroborated by a study conducted by Fifika et al., which revealed that carbon ion beam treatment had a similar effect on the growth of rice seeds [128]. It was further discovered that the inhibitory effect of low-energy Ar ion beams on plant seedlings may be caused by increased ROS production [44]. Chen et al. observed that when *Medicago truncatula* was exposed to low-energy Ar^+^ ion beam radiation, ROS production was elevated and the activity of antioxidant systems (SOD, CAT, and POD) was inhibited, resulting in a significant suppression of seed germination and seedling establishment, as well as decreased primary root and primary stem length [45]. However, these effects were mitigated by dimethylsulfoxide (DMSO) treatment [45]. Zhang et al. demonstrated that the inhibition of *Arabidopsis* seedling root growth by low-energy N^+^ beam was caused by excessive ROS production in root meristematic tissues, which interfered with cellular activity, leading to reduced meristematic cell viability and inhibited meristematic cell division [7]. In addition, *Arabidopsis RBOHs* (respiratory burst oxidase homologs) genes are involved in encoding NADPH oxidases [129]. Studies have reported that *Rboh D* and *Rboh F* genes play an important role in abscisic acid (ABA)-induced ROS production and are involved in ABA inhibition of root elongation [130]. Zhang et al. found that the transcript levels of *Atrbohs*, which include *AtrbohB*, *AtrbohC*, *AtrbohD*, and *AtrbohF*, were significantly enhanced in *Arabidopsis* treated with low-energy N^+^ beam [7]. Chen et al. demonstrated that ion beam treatment could enhance the expression of ABA biosynthetic genes, resulting in an increase in ABA content in rice [131]. Therefore, it is hypothesized that ion beam injection, as abiotic stress, may trigger the accumulation of ABA, which induces the expression of *Rboh D* and *Rboh F* genes, leading to the overproduction of ROS and thus participating in the regulatory process of ABA on plant root cells (Figure 2).

## 4. The Function of ROS in Plant Response to Plasma

Plasma, which is a partially or entirely ionized gas, can be ignited at low atmospheric conditions and is composed of charged particles (electrons, positive and negative ions), neutral species (atomic and/or molecular radicals and non-radicals), electric fields, and photons [8]. In the field of plant science, research has focused on the utilization of plasma in plant physiology and the standardization of parameter processing [132]. Numerous studies have demonstrated that plasma can enhance seed germination and promote seedling growth in a wide range of plant species (including *Arabidopsis*, tomato, wheat, oats, soybeans, poppy, hemp, rape, lentils, and mung beans) [47,133,134,135,136,137]. The common types of plasma are atmospheric cold plasma (ACP) [138], cold plasma (CP) [139], plasma-activated water (PAW) [140] and atmospheric pressure cold plasma (APCP) [141]. These plasmas can produce ROS themselves or promote the production of ROS in plants, thus influencing the growth and development of plants.

### 4.1. Plasma May Induce Seed Germination via ROS

**Figure 3:**
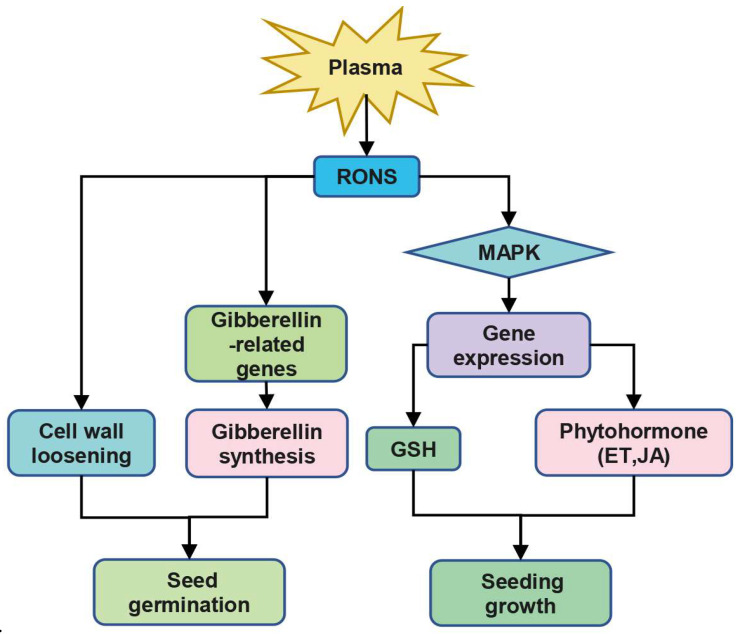
A simple mechanism for plant response to plasma. RONS, reactive oxygen and nitrogen species; MAPK, mitogen-activated protein kinase; GSH, glutathione; ET, ethylene; JA, jasmonic acid.

It has been suggested that reactive oxygen and nitrogen species (RONS) play a major role in the plasma-treatment of plants. Furthermore, the production of ROS from plasma is thought to be a key factor in the enhancement of seed germination (Figure 3) [142]. These ROS include O_2_^•−^, H_2_O_2_, and O_3_, among others. However, the exact mechanisms by which ROS affect seed germination remain unclear. One hypothesis suggests that water is essential for the uptake of ROS into the seed cell layer during swelling, which then leads to an increase in seed respiration and the subsequent oxidation of sugars, releasing metabolic energy in the form of ATP [143]. Thus, it is believed that ROS are involved in the respiratory pathway and are of great importance. Another hypothesis suggests that external ROS are detected and sensed by cells in the seeds, triggering signal transduction from the outer layers. Hydrogen peroxide is one of the active species detected in large quantities in plasma treatment, and has been demonstrated to improve the germination of tomato and pepper seeds in relatively small amounts [46]. In *Arabidopsis*, PAW containing 17–25.5 mg L^−1^ H_2_O_2_ has a positive effect on germination [47]. During germination, H_2_O_2_ is known to regulate abscisic acid (ABA) catabolism and gibberellic acid (GA) biosynthesis [144]. Endogenous H_2_O_2_ levels increase when seeds are exposed to external H_2_O_2_, resulting in various oxidative pathways such as carbonylation and lipid peroxidation [145]. Ozone, another major ROS, has been shown to enhance seed germination and induce seed protein expression [146,147]. Treatment with 200 ppm ozone generated by a plasma device for 10 min modified the coat of *Arabidopsis* seeds [48]. Our previous research revealed that the short-term plasma treatment of seeds caused plasma-induced production of O_2_^•−^, ·OH and NO, breaking seed dormancy and thus increasing seed germination; however, high concentrations of H_2_O_2_ caused oxidative damage to seeds treated with prolonged plasma, thus inhibiting seed germination [49]. Similarly, Whitaker et al. found that ·OH and O_2_^•−^ were involved in relieving seed dormancy, while H_2_O_2_ inhibited seed germination [50]. Plasma treatment of plant seeds produces ROS in the seeds, which regulates the expression of gibberellin-related genes, induces gibberellin synthesis, and affects seed germination [50]. Mujahid et al. also demonstrated that it is possible that ·OH causes cell wall loosening, which in turn promotes seed germination [51].

### 4.2. Plasma May Promote Seedling Growth via ROS

Numerous studies have shown that plasma has a stimulatory effect on plant growth [49,51,148,149]. However, the exact mechanism of how plasma induces plant growth is still unclear. Our research on *Arabidopsis* seedlings at the molecular and genetic levels suggests that ROS may be involved in the signal transduction process, thus promoting plant growth (Figure 3).

Research has demonstrated that the effect of plasma treatment on plant growth is dose-dependent [150,151]. Past studies have established that short-term APCP (Atmospheric Pressure Cold Plasma) treatments (0.5, 1, and 3 min) have a positive impact on the growth of *Arabidopsis* seedlings, while longer APCP treatments (5 and 10 min) had the opposite effect [30]. A recent study has revealed that APCP can produce large amounts of RONS in liquid, and that these component-rich RONS can alter solution properties and chemical composition. These changes can act as either stimulants or oxidative stress to regulate the growth of *Arabidopsis* seedlings [152]. It has been determined that short-term APCP treatments produce moderate RONS, which can be considered an oxidative stimulus to increase the activity of the antioxidant system and promote seedling growth. Conversely, prolonged APCP treatment produces excessive RONS that disrupt cellular redox homeostasis, leading to oxidative stress and inhibiting seedling growth [152]. Additionally, evidence has shown that APCP treatment can also have a long-term positive effect on plant growth, likely due to ROS-triggered induction [28]. Furthermore, APCP treatment has been found to stimulate an increase in intracellular ROS (O_2_^•−^, ·OH, H_2_O_2_) in plants [30]. RNA-seq analysis has demonstrated that GSH-related *ROXY3* was significantly up-regulated after APCP treatment, suggesting that *ROXY3* may play an important role in APCP-enhanced *Arabidopsis* seedling growth by mitigating APCP-induced oxidative stress [30]. Our study identified five DEGs (*WRKY33* (*AT2G38470*), *PAD3* (*AT3G26830*), *OXI1* (*AT3G25250*), *PDF1.2 A* (*AT5G44420*) and *PDF1.2 C* (*AT5G44430*)) belonging to the MAPK signaling pathway, suggesting that APCP may promote the growth of *Arabidopsis thaliana* by regulating this pathway. RONS (especially H_2_O_2_ and NO) produced by CP have been found to affect seed germination, plant growth and development, and stress resistance [52,53]. *CYP735A2* and *GA2ox8* were upregulated in our study and are associated with zeatin (cytokinin) and diterpene (GA) biosynthetic pathways, respectively. Additionally, nine genes (*AT5G64750*, *AT4G06746*, *AT2G47520*, *AT2G05520*, *AT3G15356*, *AT5G07100*, *AT3G04070*, *AT1G06160 and AT3G23230*) were downregulated and associated with ethylene synthesis, while four genes (*AT2G38240*, *AT5G05600*, *AT3G48520*, *AT2G02990*) were downregulated and linked to JA synthesis [30]. Thus, it is hypothesized that APCP-derived inducers (especially ROS) may act as an effective inducer to regulate the expression of GSH and phytohormone genes through the MAPK signaling pathway, thereby promoting the growth of *Arabidopsis* seedlings.

## 5. Conclusions

Plants respond to different forms of radiation in a variety of ways, some of which are detrimental, such as lipid peroxidation, inhibition of seedling growth, reduction of photosynthetic efficiency, and in extreme cases, even cell death. However, radiation can also be used to positively affect plants, by increasing resistance, and promoting the growth of adult plants.

When exposed to radiation, plants may initiate a signal transduction pathway via the production of ROS such as O_2_^•−^, H_2_O_2_,·OH, O_3_, and singlet oxygen (1O_2_). The plant then activates its antioxidant system to counteract the effect of the ROS and maintain its own ROS level in balance. It has been observed that plants respond differently to the same kind of radiation. Under the same radiation conditions, it may cause damage to the plant but it may also be beneficial for its growth. Therefore, for different varieties of plants, it is necessary to explore more specialized radiation conditions, analyze the relationship of radiation-induced signaling pathways at the level of ROS molecules, and use radiation to improve crops. It has been found that UV, ion beam, and plasma radiation can promote seed germination, while UV radiation can affect plant photosynthesis by inducing ROS. Additionally, ion beam radiation-induced ROS may be associated with plant stress resistance factors. Moreover, ROS has been found to have different regulatory effects in other aspects of the plant. For example, Huang et al. suggested that the regulation of ROS homeostasis plays a critical role in many processes, from apical meristem maintenance to nascent shoot initiation [18]; Marzol et al. found that, in *Arabidopsis thaliana*, growth hormone promotes the expression of a series of ROS-related genes by activating the expression of *RSL4*, thereby regulating root hair cell elongation, indicating that ROS also play an important role in root hair development [153]. Thus, further research is needed to investigate the link between ROS and radiation and plants.

## Figures and Tables

**Table 1:** Major advances in the role of ROS in plants in response to different radiation.

Radiation	Plants	ROS	Major Advances	Reference
Low-energy N^+^ beam	*Arabidopsis thaliana*	ROS	Interfere with cellular activity, leading to reduced meristematic cell viability and inhibited meristematic cell division	[7]
Carbon ion beam	*Arabidopsis thaliana*	ROS	Promote seedling growth	[15]
Atmospheric pressure cold plasma (APCP)	*Daucus carota*	ROS	Have a long-term positive effect on growth	[28]
UV-B	*Glycine max*	H_2_O_2_	Mediate isoflavones synthesis	[29]
Atmospheric pressure cold plasma (APCP)	*Arabidopsis thaliana*	ROS	Regulate the expression of GSH and phytohormone genes	[30]
UV	*Daucus carota*	ROS	Activate ethylene (ET) and jasmonic acid (JA) biosynthesis	[31]
UV-B	*Raphanus sativus*	H_2_O_2_	Mediate anthocyanin biosynthesis	[32]
UV-B	*Zea mays*	ROS	Reduce PS II photochemical efficiency	[33]
UV-B	*Vicia faba*	·OH	May be one of the mechanisms of UV-B-induced damage	[34]
UV-B	*Eucalyptus globulus* and *Olea europea*	ROS	ROS was associated with a decrease in pigmentation	[35,36]
UV-B	*Pisum sativum*, *Cucumis sativus* and *Hordeum vulgare*	ROS	Degrade Rubisco via proteolytic degradation of large subunits (LSU)	[37,38,39]
UV-B	*Arabidopsis thaliana*	O_2_^•−^	Induce the expression of *PDF1.2*	[40]
UV-B	*Arabidopsis thaliana*	H_2_O_2_	Increase the expression of *PR-1* but inhibits *Lhcb*	[40]
Carbon ion beam	*Arabidopsis thaliana*	ROS	Increase heat tolerance	[41]
Carbon ion beam	*Arabidopsis thaliana*	ROS	Increase cold tolerance	[42]
^12^C^6+^-ion beam	*Triticum aestivum*	H_2_O_2_, O_2_^•−^	Improve disease resistance	[43]
Ar-ion beam	*Arabidopsis thaliana*	ROS	Inhibit seeding growth	[44]
Ar^+^ ion beam	*Medicago truncatula*	ROS	Suppress seed germination and seedling establishment, as well as decrease primary root and primary stem length	[45]
Cold air plasma	*Solanum lycopersicum* and *Capsicum annum*	H_2_O_2_	Improve seed germination	[46]
Plasma-activated water (PAW)	*Arabidopsis thaliana*	H_2_O_2_	A positive effect on germination	[47]
Plasma	*Arabidopsis thaliana*	O_3_	Modify the coat of seeds	[48]
Plasma	*Arabidopsis* and *Bidens pilosa*	O_2_^•−^, ·OH	Break seed dormancy and thus increasing seed germination	[49,50]
Plasma	*Arabidopsis* and *Bidens pilosa*	H_2_O_2_	Inhibit seed germination	[49,50]
Plasma	*Bidens pilosa*	ROS	Regulate the expression of gibberellin-related genes	[50]
Cold plasma (CP)	*Vitis vinifera*	·OH	Cause cell wall loosening, which in turn promotes seed germination	[51]
Cold plasma (CP)	*Solanum lycopersicum* and *Raphanus sativus*	ROS	Affect seed germination, plant growth and development, and stress resistance	[52,53]

**Table 2:** Reaction mechanisms of major reactive oxygen species (ROS) scavenging enzymatic and non-enzymatic antioxidants.

Antioxidants	Reactions Catalyzed
Catalase (CAT)	2H_2_O_2_ → 2H_2_O + O_2_
Peroxidase (POD)	2H_2_O_2_ → 2H_2_O + O_2_
Ascorbate peroxidase (APX)	H_2_O_2_ + AsA → 2H_2_O + MDHA
Glutathione reductase (GR)	GSSG + NADPH + H+ → GSH + NADP+
Superoxide dismutase (SOD)	2O_2_ ^•−^ + 2H+→ O_2_ + H_2_O_2_
Ascorbic acid (ASA)	Scavenges O_2_ ^•–^, H_2_O_2_, ·OH, and ^1^O_2_
Glutathione (GSH)	Scavenges H_2_O_2_, ·OH, and ^1^O_2_
Flavonoids	Scavenges O_2_ ^•–^, H_2_O_2_, and ^1^O_2_

## Data Availability

Not applicable.
